# Patient portal adoption and use by hospitalized cancer patients: a retrospective study of its impact on adverse events, utilization, and patient satisfaction

**DOI:** 10.1186/s12911-018-0644-4

**Published:** 2018-07-27

**Authors:** Duaa Aljabri, Adrian Dumitrascu, M. Caroline Burton, Launia White, Mahmud Khan, Sudha Xirasagar, Ronnie Horner, James Naessens

**Affiliations:** 1 Department of Health Sciences Research, Mayo Clinic, Jacksonville, FL USA; 2 Division of Hospital Internal Medicine, Mayo Clinic, Jacksonville, FL USA; 3 Division of Biomedical Statistics and Informatics, Mayo Clinic, Jacksonville, FL USA; 4 Department of Health Sciences Research, Mayo Clinic, Rochester, MN USA; 5 Department of Health Services Policy and Management, University of South Carolina, Columbia, SC USA

**Keywords:** Adverse events, Cancer, Hospitalization, Portal, Satisfaction, Utilization

## Abstract

**Background:**

Portal use has been studied among outpatients, but its utility and impact on inpatients is unclear. This study describes portal adoption and use among hospitalized cancer patients and investigates associations with selected safety, utilization, and satisfaction measures.

**Methods:**

A retrospective review of 4594 adult hospitalized cancer patients was conducted between 2012 and 2014 at Mayo Clinic in Jacksonville, Florida, comparing portal adopters, who registered for a portal account prior to hospitalization, with nonadopters. Adopters were classified by their portal activity during hospitalization as active or inactive inpatient users. Univariate and several logistic and linear regression models were used for analysis.

**Results:**

Of total patients, 2352 (51.2%) were portal adopters, and of them, 632 (26.8%) were active inpatient users. Portal adoption was associated with patients who were young, female, married, with higher income, and had more frequent hospitalizations (*P* < .05). Active inpatient use was associated with patients who were young, married, nonlocals, with higher disease severity, and were hospitalized for medical treatment (*P* < .05). In univariate analyses, self-management knowledge scores were higher among adopters vs nonadopters (84.3 and 80.0, respectively; *P* = .01) and among active vs inactive inpatient users (87.0 and 83.3, respectively; *P* = .04). In regression models adjusted for age and disease severity, the association between portal behaviors and majority of measures were not significant (*P* > .05).

**Conclusions:**

Over half of our cancer inpatients adopted a portal prior to hospitalization, with increased adoption associated with predisposing and enabling determinants (eg: age, sex, marital status, income), and increased inpatient use associated with need (eg: nonlocal residence and disease severity). Additional research and greater effort to expand the portal functionality is needed to impact inpatient outcomes.

## Background

Two decades ago, the National Academy of Medicine (formerly the Institute of Medicine) recommended implementation of electronic health records to improve quality of care in the United States [1]. Since then, health information technologies have been rapidly adopted, with a focus on providers rather than patients. In 1996, the Health Insurance Portability and Accountability Act legally allowed patients to access their own clinical records. However, record retrieval fees, illegible handwriting, and time delays hindered accessibility [2]. An additional challenge is the fragmented health system with many independently owned and operated health care service locations [3–5]. An integrated information system that aggregates and offers updated health information to patients through a single access point was needed. In 2009, the Health Information Technology for Economic and Clinical Health Act incentivized clinicians to provide patients with electronic access to clinical records through meaningful use rules, administered by the Centers for Medicare & Medicaid Services [6]. This incentive program remains the principal driver of patient portal development by funding nearly $30 billion in provider incentives to encourage appropriate use [7, 8]. Investigations where information access was offered via patient portals in the outpatient settings showed encouraging positive effects in patient satisfaction and self-management behaviors [4, 9–18]. However, providing patients access to information is important not only in home and outpatient settings, but also when patients are hospitalized [19].

When patients are able to see their own health information during the hospital stay, they become more informed, empowered to ask questions, and gain ownership of their health care [20, 21]. Despite daily bedside rounds, important patient informational needs may not be met due to the cost of reviewing tailored information with each patient individually [22]. Thus, the portal technology may provide opportunity for inpatients to meet informational needs, facilitate awareness, and improve understanding of their care during hospitalization and after discharge [2, 23]. Meeting informational needs could reduce uncertainties surrounding the care process, reduce information asymmetry between patients and providers, promote shared decision-making, and increase patient self-management and adherence to care [24, 25].

Unfortunately, assessments of patient portal use among hospitalized cancer patients are limited [8, 26–30]. For many patients, the hospital is a challenging and intimidating setting, compounded by unmet information needs and limited patient engagement [24, 31]. The rapid dynamic and pace of clinical care, changing medical teams, reliance on verbal communication, and absence of an established relationship with the care providers further challenge patients’ effective participation in their own care [32, 33]. Additional affective and emotional challenges are faced by inpatients with cancer due to the nature of their disease, frequently uncertain outcomes of treatments, and the need to understand their multiple active conditions to make treatment decisions [34, 35]. In a study of breast cancer patients, those who desired an active role in treatment decision making also desired detailed information of their diagnosis, treatment procedures, and alternatives [36]. Similar information needs were vital to gynecologic and colorectal cancer patients who felt that information about the likelihood of cure, spread of disease, and treatment options were priorities for decision making [37]. Providing clinical information through patient portals may have the potential to transform the patient-physician relationship and help patients to become active in their disease management [38]. Recent documentation on hospital-based patient portals is encouraging [39–42]. Creber et al. published a protocol for developing a personalized inpatient portal at an urban academic medical center to improve cardiology inpatients engagement [2]. Greysen et al. conducted pilot interviews showing patients’ enthusiasm for a tablet application that provides health information during their inpatient stay [43]. Vawdrey et al. assessed the patient-perceived efficacy of tablets to improve cardiothoracic surgery patients’ engagement in care, showing a favorable response regarding usability of the application [19]. Several other studies assessed the feasibility of web-based applications to increase patient engagement in both pediatric and adult care [44–46]. Yet, the evaluation of patient portals among cancer inpatients is still limited, a knowledge gap addressed by this study. We hypothesized that patient adoption of a portal and active use during a hospital stay may be associated with greater patient safety, postdischarge care utilization and satisfaction, similar to outpatient settings. According to Karahanna et al., adoption and continued use represent different behaviors [47]. Adoption is the initial enrollment and signifies receptivity to the portal, while usage represents active engagement, continued use after adoption. Therefore we distinguish between these 2 behaviors and evaluate them separately. Our specific aims were to 1) identify the key patient factors predicting adoption and active inpatient use behaviors, and 2) examine the association between portal use behaviors and adverse events, postdischarge utilization (emergency visits and readmissions), and selected patient satisfaction measures (self-health management knowledge and satisfaction with the overall hospital experience).

## Methods

### Study setting and description of the portal

The site of the study was Mayo Clinic, Jacksonville, Florida (MCF), a large nonprofit, specialized tertiary care practice and medical research center with more than 1.3 million domestic and international patients seen each year. Physicians are salaried, not linked to care volume, thus reducing monetary incentives in patient treatment. MCF contracted with Cerner Solutions (Cerner Corp) to implement the patient portal and integrate it with the system-wide electronic health record in 2010. When patients schedule an appointment at MCF, they are invited to register for a portal account and are provided with information on why and how to register. With each appointment reminder, patients receive a reminder message to register for the portal. Portal invitations are also offered in all outpatient waiting areas and displayed on electronic screens around the clinic.

Once registered, patients’ are able to access informational functions, such as viewing lab results, current medications, allergies, and diagnostic reports from clinic visits and hospitalizations, and administrative functions, such as paying bills, processing prescription refills, and coordinating appointments. A Continuity of Care Document, a complete summary of patient current health status and history, is also available to view, download, or forward to physicians at other hospitals. Additional information on MCF patient portal is documented elsewhere [48]. Although the portal is designed for outpatients, some functions are applicable to inpatient health information needs during the hospital stay. Hospitalized patients potentially have time to access the portal when they are not occupied with diagnostic testing or other activities [49]. For example, the portal gives inpatients real-time access to laboratory results, admission notes, consultation reports, and surgical notes, to view on their own time and between bedside rounds. This functionality potentially facilitates patient communication and interaction with the health care team during their stay, and empowers the patient to be more attentive toward errors in documentation [20]. In addition, the medication function provides patients with information on the type and purpose of their medications, including in-hospital medication intake, which could enable patients to ask questions, review for accuracy, or report medication discrepancies [50, 51]. Before home discharge, a discharge summary and discharge instructions is uploaded to the portal, giving patients time to review closely and ensure their understanding of home self-management instructions. While the development of portal functionality for inpatients is in early stages, the offered content may still help patients become more activated and improve postdischarge care.

### Study design and participants

This was a retrospective review of patients satisfying the following criteria: 1) adults 18 years of age or older, 2) cancer as a primary or secondary diagnosis at time of hospitalization identified through the *International Classification of Diseases, Ninth Revision* (ICD-9) codes, and 3) admitted to MCF between August 1, 2012, and July 31, 2014 (*N* = 4594). Per the unified theory of acceptance of use of technology, user acceptance and intention to use a technology is followed by actual use [52]. Therefore, we included the first hospitalization where a portal account had been established prior to admission to examine consequent inpatient use. If the patient had not established a portal account prior to any admission, then the first hospitalization in the study period was selected. Patients who had a portal account prior to admission were defined as *adopters*, and those without a portal account were *nonadopters*. Among adopters, inpatients who logged in their portal during the hospital stay were *active inpatient users* and those who never logged in were referred to as *inactive inpatient users*. The study was approved by the Mayo Clinic Institutional Review Board.

### Study model

**Fig. 1:**
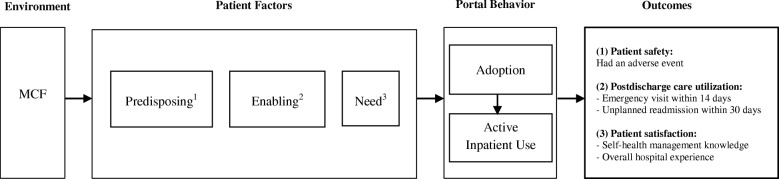
Study Theoretical Model Derived from Andersen’s Model of Healthcare Utilization. 1 Predisposing factors: age, sex, and race. 2 Enabling factors: marital status, employment status, health insurance type, and income. 3 Need factors: geographic area of residence, comorbidities, and frequency of hospitalizations. Additional need factors related to the admission: MSDRG type and APRDRG disease severity weight. APRDRG indicates All Patients Refined Diagnostic Related Group; MSDRG, Medicare Severity-Diagnosis Related Group

Our study was informed by Andersen’s Model of Healthcare Utilization [53]. The model was initially developed in 1968 to understand health services use and later revised to include consumer satisfaction and dimensions of health status [54]. Shortly after the model was developed, health services use was portrayed as a health behavior influenced by multiple factors [55]. According to the World Health Organization, health behavior is defined as “any activity undertaken by an individual, regardless of actual or perceived health status, for the purpose of promoting, protecting or maintaining health, whether or not such behavior is objectively effective towards that end” [56]. Because the portal is a tool to maintain and promote health, we considered portal adoption and use as health behaviors that could be studied using Andersen’s model. As shown in Fig. 1, we examined the influence of patients’ characteristics in three major components: predisposing, enabling, and need factors, on portal adoption and use behaviors.

### Measures

#### Environment and patient characteristics

In this study, we assumed that all study participants had a common environmental context, as all patients in MCF received their care in the same structure. Predisposing determinants included age, sex, and race. Enabling determinants included marital status, employment status, health insurance type, and median income in the residential ZIP code less than Florida’s state median income, a surrogate for socioeconomic status. Need factors included geographic area of residence, comorbidities, and frequency of hospitalizations in the study period. Additional need determinants related to the hospital admission included patient’s disease severity weight as measured by the 3 M All Patients Refined Diagnosis Related Groups (APDRG) classification system, and whether the hospitalization was for medical or surgical treatment, based on the Medicare Severity-Diagnosis Related Group (MSDRG) codes [57].

Demographic data were extracted from the patient electronic health records. The ZIP code median income was obtained from the 2006–2010 American Community Survey and matched to the patient sample at the ZIP code level [58]. A count of comorbidities included in Charlson Comorbidity Index during the 12 months prior to hospitalization was documented [59].

#### Patient safety, utilization, and satisfaction

We examined selected patients’ measures to investigate associations with portal use. For patient safety, we studied the occurrence or otherwise of provider-reported, in-hospital, adverse events, such as falls, accidental self-injuries, or other events related to the surgery, vascular, equipment or devices, medication, or skin events, obtained from quality management services. For postdischarge utilization, we examined the occurrence of emergency department visits within 14 days and unplanned readmissions within 30 days, both obtained from the hospital records. We measured patient satisfaction by obtaining data from the Hospital Consumer Assessment of Healthcare Providers and Systems (HCAHPS) survey. The survey is a validated instrument used since 2006 to assess patients’ perspectives of hospital care, and distributed to a random sample of discharged patients between 2 days and 6 weeks after discharge [60]. While the survey included many important questions, we selected the relevant items with highest response. We measured *patient self-health management knowledge* with 2 questions: “When I left the hospital, I had a good understanding of the things I was responsible for in managing my health”, and “When I left the hospital, I clearly understood the purpose for taking each of my medications”, and measured *overall hospital satisfaction* with 1 question: “Using any number from 0 to 10, where 0 is the worst hospital possible and 10 is the best hospital possible, what number would you use to rate this hospital during your stay” [61]. Responses were transformed and averaged, resulting in a 0 to 100 linear-scaled score.

### Data analysis

We described the characteristics of cancer patients according to their portal adoption and inpatient activity behaviors, and examined differences between groups using Pearson χ^2^ and Wilcoxon nonparametric tests. Multivariate regression models were conducted to predict factors associated with portal adoption and active inpatient use, as well as to examine the association between selected outcomes and portal behaviors. All analyses were conducted in SAS Version 9.4 (SAS Institute Inc., Cary, North Carolina, USA), and significance was defined as *P* < .05.

## Results

### Participants, adopters, and active inpatient users

Of the 4594 study-eligible hospitalized patients with cancer, 2352 (51.2%) had a portal account prior to admission (ie, adopters), of whom 632 (26.8%) used the portal account during their hospital stay (ie, active inpatient users). Patient characteristics at admission were reported in Table 1. Significant differences in patient characteristics were present among portal adoption and inpatient use behaviors (Table 2). Adoption was influenced by a majority of predisposing and enabling factors, such as age, sex, race, marital status, employment status, income, and type of health insurance. While active inpatient use was similarly influenced by predisposing and enabling factors, such as age, race, and marital status, we found greater influence associated with need, such as having greater disease severity, being nonlocal, and admitted for medical rather than surgical treatment.

**Table 1:** Sample Baseline Characteristics by Portal Behavior^a^

Characteristics		Adopters (*n* = 2,352)	Nonadopters (*n* = 2,242)	*P* value	Active Inpatient Users (*n* = 632)	Inactive Inpatient Users (*n* = 1,720)	*P* value
Age group (years)	Mean (SD)	62.3 (14.0)	65.4 (14.8)	<.01^b^	60.2 (14.3)	63.0 (13.8)	<.01^b^
	≤44	259 (11.0)	191 (8.5)	<.01^c^	82 (13.0)	177 (10.3)	<.01^c^
	45-54	339 (14.4)	281 (12.5)		106 (16.8)	233 (13.5)	
	55-64	632 (26.9)	480 (21.4)		185 (29.3)	447 (26.0)	
	65-74	702 (29.8)	652 (29.1)		166 (26.3)	536 (31.2)	
	75-84	339 (14.4)	454 (20.2)		80 (12.7)	259 (15.1)	
	≥85	81 (3.4)	184 (8.2)		13 (2.1)	68 (4.0)	
Sex	Female	1,148 (48.8)	1,055 (47.0)	.25^c^	295 (46.7)	852 (49.5)	.22^c^
	Male	1,204 (51.2)	1,188 (53.0)		337 (53.3)	869 (50.5)	
Race	African American	121 (5.2)	273 (12.4)	<.01^c^	18 (2.9)	103 (6.1)	<.01^c^
	White	2,120 (91.3)	1847 (84.0)		575 (91.9)	1,545 (91.2)	
	Other	80 (3.4)	78 (3.5)		33 (5.3)	47 (2.8)	
Marital status	Married	1,786 (75.9)	1,454 (64.9)	<.01^c^	506 (80.1)	1,280 (74.4)	<.01^c^
	Single/divorced/widowed	566 (24.1)	788 (35.1)		126 (19.9)	440 (25.6)	
Employment status	Employed	739 (37.7)	547 (28.9)	<.01^c^	206 (38.1)	533 (37.5)	.79^c^
	Retired	836 (42.6)	935 (49.4)		208 (38.5)	628 (44.2)	
	Not employed/disabled	386 (19.7)	411 (21.7)		126 (23.3)	260 (18.3)	
Income	Below Florida median income	645 (28.3)	803 (37.2)	<.01^c^	167 (26.5)	478 (27.8)	.54^c^
	At or above Florida median income	1,707 (71.7)	1,439 (64.1)		465 (73.5)	1,242 (72.2)	
Health insurance	Commercial insurance/self pay	1,145 (48.7)	815 (36.4)	<.01^c^	339 (53.6)	806 (46.9)	<.01^c^
	Medicare/Medicaid/other government assistance	1,207 (51.3)	1,427 (63.6)		293 (46.4)	914 (53.1)	
Geographic area of residence	Nonlocal	521 (22.2)	493 (22.0)	.89^c^	166 (26.3)	335 (20.6)	<.01^c^
	Local	1,831 (77.8)	1,749 (78.0)		466 (73.7)	1,365 (79.4)	
Comorbidity ^d^	None	1,115 (47.4)	1,052 (46.9)	.58^c^	289 (45.7)	826 (48.0)	.32^c^
	One or more	1,237 (52.6)	1,190 (53.1)		343 (54.3)	894 (52.0)	
Comorbidity type^e^	Congestive heart failure	145 (6.2)	171 (7.6)	.05^c^	51 (8.1)	94 (5.5)	.02^c^
	Peripheral vascular disease	322 (13.7)	338 (15.1)	.18^c^	83 (13.1)	239 (13.9)	.63^c^
	Cerebrovascular disease	165 (7.0)	224 (10.0)	<.01^c^	42 (6.6)	123 (7.2)	.67^c^
	Chronic pulmonary disease	262 (11.1)	256 (11.4)	.77^c^	61 (9.7)	201 (11.7)	.16^c^
	Mild liver disease	432 (18.4)	291 (13.0)	<.01^c^	129 (20.4)	303 (17.6)	.12^c^
	Diabetes mellitus	392 (16.7)	351 (15.7)	.35^c^	115 (18.2)	277 (16.1)	.23^c^
	Moderate/severe renal disease	196 (8.3)	179 (8.0)	.66^c^	53 (8.4)	143 (8.3)	.96^c^
	Moderate/severe liver disease	121 (5.1)	86 (3.8)	.03^c^	49 (7.8)	72 (4.2)	<.01^c^
Admission type based on MSDRG	Surgical	1,504 (63.9)	1,315 (58.7)	<.01^b^	348 (55.1)	1,156 (67.2)	<.01^b^
	Medical	848 (36.1)	927 (41.3)		284 (44.9)	564 (32.8)	
Frequency of hospitalizations, mean (SD)		1.8 (1.5)	1.4 (1.1)	<.01^b^	2.0 (1.7)	1.7 (1.4)	<.01^b^
APRDRG weight, mean, (median, SD)		2.5 (1.5, 2.9)	2.3 (1.5, 2.3)	.24^b^	3.1 (1.9, 3.6)	2.3 (1.4, 2.6)	<.01^b^

*Abbreviations: APRDRG* All Patients Refined Diagnosis Related Group, *MSDRG* Medicare Severity-Diagnosis Related Group

^a^Data are reported as No. (%) for count variables and mean (SD) for continuous variables

^b^Wilcoxon nonparmetric

^c^Pearson χ^2^ test

^d^Comorbidity groups are not mutually exclusive as a patient may have more than 1 comorbidity diagnosis

^e^Comorbidity type was reported for diseases with > 5% of patients

**Table 2:** Logistic Regression Analysis Showing the Predictors of Portal Behaviors

Factors	Characteristics		Odds Ratio (95% CI)^a^	
			Adoption^b^	Active Inpatient Use^c^
Predisposing	Age	44-	**1.42 (1.03, 1.95)**	**1.89 (1.16, 3.06)**
		45-54	1.10 (0.82, 1.47)	**1.73 (1.10, 2.72)**
		55-64	1.22 (0.95, 1.57)	**1.48 (1.00, 2.19)**
		65-74 (reference)	1.00	1.00
		75-84	**0.71 (0.57, 0.87)**	1.08 (0.75, 1.56)
		85+	**0.36 (0.24, 0.54)**	0.77 (0.34, 1.75)
	Sex	Male (reference)	1.00	1.00
		Female	**1.26 (1.10, 1.45)**	0.97 (0.78, 1.21)
	Race	White (reference)	1.00	1.00
		African American	**0.34 (0.27, 0.45)**	**0.51 (0.29, 0.89)**
		Others	0.84 (0.58, 1.23)	1.39 (0.82, 2.37)
Enabling	Marital status	Divorced/single/widowed (reference)	1.00	1.00
		Married	**1.60 (1.37, 1.87)**	**1.49 (1.14, 1.94)**
	Employment	Employed (reference)	1.00	1.00
		Retired	1.04 (0.83, 1.30)	1.04 (0.75, 1.46)
		Not employed/disabled	**0.70 (0.57, 0.86)**	1.04 (0.77, 1.40)
	Health insurance	Commercial insurance (reference)	1.00	1.00
		Medicaid/Medicare	**0.76 (0.61, 0.95)**	1.07 (0.77, 1.50)
	Income	Below FL median income (reference)	1.00	1.00
		At or above FL median income	**1.39 (1.20, 1.60)**	1.10 (0.87, 1.39)
Need	Geographic area of residence	Local (reference)	1.00	1.00
		Nonlocal	1.13 (0.96, 1.33)	**1.34 (1.04, 1.71)**
	Comorbidities	None (reference)	1.00	1.00
		1+	1.05 (0.91, 1.21)	0.97 (0.77, 1.22)
	MSDRG type ^*d*^	Surgical (reference)	-	1.00
		Medical	-	**2.17 (1.68, 2.78)**
	Frequency of hospitalizations		**1.43 (1.33, 1.55)**	1.08 (0.99, 1.19)
	APRDRG weight ^*d*^		-	**1.13 (1.09, 1.17)**

*Abbreviations: APRDRG* All Patients Refined Diagnosis Related Group, *MSDRG* Medicare Severity-Diagnosis Related Group

^a^Bold values are statistically significant at *P*<.05. Odds ratios greater than 1 imply increased chance for behavior; less than 1 imply decreased chance for behavior

^b^Predictors for adoption: age, sex, race, marital status, employment status, income, health insurance type, and frequency of hospitalizations

^c^Predictors for active inpatient use: age, race, marital status, geographic area of residence, APRDRG weight, and MSDRG type

^d^Variables related to the hospital admission were not examined among adopters as the adoption behavior was established prior to admission

### Bivariate associations of portal behaviors with adverse events, care utilization, and patient satisfaction

Bivariate associations of portal adoption with our selected measures (Table 3) showed that adopters had more emergency visits and readmissions than nonadopters, while reporting higher self-health management knowledge. Similarly, active inpatient users had more readmissions than inactive inpatient users, and marginally higher self-management scores. Logistic and linear regression analyses showed that after adjusting for age and disease severity, the association between portal behaviors and majority of our assessed measures were not significant (Table 4). Adverse events and overall hospital experience did not differ among groups in either univariate or multivariate regression analyses (*P* > .05).

**Table 3:** Adverse Events, Postdischarge Care Utilization and Patient Satisfaction Among the portal Behavior Groups

Measures	Outcomes	Adopters (*n* = 2352)	Nonadopters (*n* = 2242)	*P* value	Active Inpatient Users (*n* = 632)	Inactive Inpatient Users (*n* = 1720)	*P* value
Patient safety	Had an adverse event, No. (%)	40 (1.7)	47 (2.1)	.36^a^	13 (2.1)	27 (1.6)	.42^a^
Postdischarge care utilization	Emergency visit within 14-days of discharge, No. (%)	272 (11.6)	214 (9.5)	.03^a^	75 (11.9)	197 (11.5)	.78^a^
	30-day unplanned readmission, No. (%)	299 (12.7)	222 (9.9)	<.01^a^	96 (15.2)	203 (11.8)	.03^a^
Patient satisfaction^c^	Understand responsibilities for self-health management, mean score (SD)	87.6 (19.6)	85.5 (20.1)	.02^b^	89.1 (18.4)	87.1 (20.0)	.22^b^
	Understand the purpose for taking each medication, mean score (SD)	90.0 (18.8)	87.8 (20.9)	.05^b^	91.8 (16.0)	89.4 (19.7)	.21^b^
	Aggregate self-health management knowledge score	84.3 (21.3)	80.0 (23.1)	<.01^b^	87.0 (19.2)	83.3 (21.9)	.05^b^
	Overall rating of the hospital stay, mean score (SD)	95.6 (9.1)	95.3 (10.3)	.75^b^	95.6 (10.5)	95.7 (8.6)	.56^b^

^a^Pearson χ^2^ test

^b^Wilcoxon nonparametric

^c^Satisfaction survey was distributed to a random sample of discharges; thus, the sample size was as follows: adopters; *n* = 788, nonadopters; *n* = 646, active inpatient users; *n* = 205, and inactive inpatient users; *n* = 577

**Table 4:** Association Between Patient Outcomes and Portal Behaviors: Results From Regression Models

	Independent Variables			
	Active Inpatient Users vs Nonadopters		Inactive inpatient Users vs Nonadopters	
Dependent Variables^a^	OR (95% CI)	*P* value	OR (95% CI)	*P* value
[adverse events = yes]	0.76 (0.40, 1.45)	.41	0.71 (0.44–1.16)	.17
[emergency visits = yes]	1.28 (0.97, 1.70)	.08	1.23 (1.00–1.51)	.08
[readmissions = yes]	1.60 (1.23, 2.08)	<.01	1.21 (0.99–1.48)	.06
Dependent Variables^a^	Beta Coefficient	*P* value	Beta Coefficient	*P* value
Self-health management knowledge	2.18	.07	1.15	.17
Overall hospital experience	0.16	.83	0.28	.60

^a^Five regression models were conducted adjusting for age and disease severity. Logistic regression was used for adverse events, emergency visits, and readmissions. Linear regression was used for self-health management knowledge and overall hospital experience scores

## Discussion

To date there remains a gap in the literature evaluating the use of inpatient portals among cancer patients. This study provides important information to clinicians, administrators, and researchers, on the key patient determinants associated with portal adoption and use. Prior studies reported significant interest in patient portals among oncology populations [28, 29, 62]. Yet, to our knowledge, this is the first study to examine portal use in a large inpatient oncology cohort. In this sample, we found that portal adoption and use during hospitalization has reached modest levels and somewhat higher usage than published reports on inpatient portal use. Over half of our inpatient oncology population voluntarily adopted the portal before hospital admission and 27% actively used the portal during the stay. Dumitrascu et al. found that of 44.2% patients who had a portal account at the time of admission, only 20.8% accessed the portal during their stay [48]. Davis et al. found that of 34.4% registered portal patients, 23.4% used it while hospitalized [39]. Robinson et al. reported that 16% of surgical inpatients with a portal account used it while being in the hospital [63].

There were noteworthy differences in patient characteristics between adopters and nonadopters in a majority of predisposing and enabling factors. Portal adoption increased among patients who were female, married, and with higher income, and decreased among patients who were African American, unemployed, and had governmental health insurance. Interestingly, the likelihood of portal adoption was similar for patients aged 65 to 75 years as the middle-aged adults 45 to 55 years, contradicting popular beliefs that older patients were less likely to engage in health technologies [64]. Portal adoption, however, considerably decreased among patients aged over 75 years. Similar to our findings, portal use among outpatient oncology patients was reported to be greater among younger, white patients, and those with upper aerodigestive malignancy diagnosis, greater disease severity, and case complexity [8]. Among nononcology populations, a similar digital divide was reported by age groups, race/ethnicity, income, and education [65–70]. Our findings showed higher portal adoption among those with more frequent hospitalizations, which was the only notable need determinant. Other studies have reported higher interest in the portal among those with more medical problems, greater severity of illness, or higher than average clinical need [10, 15, 71, 72].

Similarly, inpatient portal use increased with younger age and being married, but more influenced with need determinants. Active access was associated with residing outside the city of Jacksonville (nonlocals); suggesting that commuting patients found health information important to view during the stay. Additionally, access was greater among those with higher disease severity and those admitted for medical rather than surgical treatment. Medical admissions for cancer patients are usually associated with investigating the origin and cause of disease, or evaluating chemo or radiation treatments, compared to surgical admissions that involve typical procedural routines and surgical recovery that may fully occupy the patient’s time in the hospital [73]. Because a cancer diagnosis is a stressful life event, patients’ information-seeking behavior was thought to become more active, possibly as a coping strategy to overcome uncertainties [27, 29, 74].

### Patient safety

Several studies have assumed that information technology systems have the potential to improve patient safety by identifying errors in medications and preventing adverse drug reactions. Yet, limited evidence exists regarding the effectiveness of a portal as a tool in reducing adverse events. One recent study by Kelly et al. found that 8% of parents with hospitalized children recognized errors in their child’s medication list after using an inpatient portal application [46]. Further optimistic views about the ability of portals to reduce errors were derived from patient participation in care, where patients could notify clinicians of their medication allergies, unexpected toxicity symptoms, and lapses in care to prevent adverse events [50, 75–77]. Among surgical inpatients who were portal users, postoperative infection was their most frequent ICD-9 code, suggesting that experiencing a safety-event may activate patients to follow up their personal health information to avoid further complications [63]. In contrast to this evidence, our study did not find an association between portal adoption or use and adverse events. Likewise, a randomized controlled trial by Weingart et al. did not find sufficient evidence to support an association between adverse drug events and portal use [51]. Earlier research reported that patient history evaluation in cancer care is more focused, providing the patient an opportunity to recall medical and medication information to prevent errors. [78, 79] In addition, most adverse events at hospitals are underreported and the events in our data were limited to those reported by providers. A new initiative within the portal that is gaining popularity and has the potential to prevent errors is the OpenNotes national movement, which invites patients to read their clinicians’ notes online and report back errors or safety concerns that, in turn, may avert mistakes from happening [80, 81]. Hence, it opens up a new possibility to engage patients as safety partners through their reported documentation errors.

### Utilization

Studies that examined the effect of portal use on subsequent utilization of health services showed mixed results [10, 67, 82]. A study using propensity score matching found no difference between portal users and nonusers on clinical service utilization [83]. Among members of Kaiser Permanente, a retrospective study in the Northwest found that patient access to an online portal was associated with decreased rates of primary care office visits and phone calls [84], whereas the opposite was found by Palen et al. where portal users had higher rates of office visits, phone encounters, after-hour clinic visits, emergency department visits, and hospitalizations [85]. The assumption was that if patients could view personal health information, they will be more aware, able to manage their health, and need less emergency service or hospitalizations. This expectation was not validated in our study, suggesting that a portal technology may be a complementary technology and does not substitute for health services needs of oncology patients. Mayer et al. reported 77.2% of cancer patients’ visits to the emergency department were due to pain, respiratory problems, and gastrointestinal issues, with 63.2% of those visits resulting in hospital admission [86]. Barbera et al. reported that 83.8% of cancer patients who died had visited the emergency department during their final 6 months of life with issues related to abdominal pain, dyspnea, pneumonia, fatigue, and pleural effusion [87]. Shapiro et al. found that those who had surgery during their index admission were 3 times more likely to be readmitted [88]. Weaver et al. examined cancer inpatients and found 48% of readmissions were within 1 to 2 days of discharge [89]. Donze et al. developed a predictive model and found that discharge from an oncology service was a significant predictor of unplanned readmission [90]. Similarly, a recent systematic review reported that comorbidities, older age, advanced disease, and index hospitalization length of hospital stay were significant predictors for readmission in oncology [91]. Thus, emergency department visits and readmissions may be influenced more by the nature of illness, treatment-related complications, and other such factors than avoidable reasons by portal use.

### Patient satisfaction

Our findings suggest limited evidence of the relationship between patient satisfaction and portal use. Self-management knowledge scores appear to be considerably higher among both adopters and inpatient users in bivariate associations; however, in regression analyses, associations with satisfaction were somewhat attenuated and no longer statistically significant. Our interpretation of results needs to be cautiously taken as they were limited by the random selection of sample surveyed and the selection of self-management questions. In addition, we have no assessment of self-health management knowledge at baseline. Therefore, the association between portal use and self-health management knowledge may be confounded.

Prior research has shown inconsistent conclusions regarding associations between portal use and patient satisfaction; with wide variability in the offered portal features, the outcomes evaluated, and the populations studied [4, 10, 14, 31]. In addition, the potential of patient portals for patients with chronic conditions is documented, but relatively nascent for cancer [92]. Among chronically ill patients, the portal showed promise for improving diabetic patients’ satisfaction with care, ability to self-manage, and adhering to treatments [93]. This has been accompanied by evidence of improved blood pressure control among people with newly diagnosed hypertension [94]. Patient portal access was also superior in general adherence and satisfaction with doctor-patient communication among patients with congestive heart failure [18, 95]. Yet, not all findings in the literature showed that patients with chronic conditions were amenable to improved outcomes with portal use [29, 96–98].

There are many potential recommendations to improve portal functions for inpatients. Hospitals often provide patients and families with standard information on disease and treatment options while being hospitalized, but that is not always enough [99]. An effective tool for awareness and self-management may include problem-solving support, regular education provision, treatment options with cost estimations that aid patient decision making, and consistent patients training on how to take responsibility for their own health [100].

It should be noted that emotional factors, such as anxiety or low self-efficacy, may dramatically influence self-management or symptom-coping behaviors [101, 102]. Of interest, some researchers suggest technology-based applications to provide recreational social supports to help patients cope with their illness. O’Leary et al. reported favorable patient perceptions toward games offered in the hospital-based portal [40]. The same was reported by Jameson et al.*,* who indicated that electronic gaming can be a positive distraction away from pain [103]. Innovative social support approaches offering recreational avenues via the portal may attract more users, which in turn, may improve self-management, symptom-coping, and quality of life [104]. Thus, greater attention is needed to improve the portal content and functionality for inpatients to improve patient outcomes.

This study has a number of limitations. There is limited generalizability given that our oncology cohort was from a single center. Technology limitations restricted our analysis; we could not examine frequency of inpatient log-ins, or distinguish if a portal activity was carried out by the patient or a delegated family member. Further, it would be interesting to understand if there was a dose-response type curve associated with portal use but information on the extent of use was not available. Post-discharge utilization measures were limited to care utilization at MCF, with no data on utilization elsewhere. Conclusions regarding patient safety and satisfaction measures were limited by the range of variable values; adverse events were uncommon, and patient satisfaction was almost uniformly high among all patients. Finally, low response to the HCAHPS resulted in a small sub-sample size to analyze satisfaction, a major limitation, but no other measures were readily available. Despite these limitations, the study uncovered determinants of adoption and use behaviors among a large sample of hospitalized cancer patients. Additionally, it adds new information to the growing body of literature on inpatient engagement using acute care portals. Future research directions should investigate the extent of inpatient portal use, incorporate inpatient-centered education materials, and improve the portal with functions that add the most value for cancer inpatients.

## Conclusions

We found that cancer patients had reached modest levels of portal adoption. While portal adoption increased with predisposing and enabling determinants (eg: age, sex, marital status, income), active inpatient use increased with need (eg: commute residence and high disease severity). While these findings should be cautiously interpreted, the study adds to the growing evidence that patient portals should be further addressed for inpatient care. Particularly, the study provides insights for the informatics research community and those interested in improving inpatient care and self-management support through technology.

## Abbreviations

HCAHPS: Hospital Consumer Assessment of Healthcare Providers and Systems
ICD-9: International Classification of Diseases, Ninth Revision
MCF: Mayo Clinic Florida
